# Supplementary material for: D-Tagatose-Based Product Triggers Sweet Immunity and Resistance of Grapevine to Downy Mildew, but Not to Gray Mold Disease
Source: Plants (Basel). 2022 Jan 23;11(3):296. doi: 10.3390/plants11030296 (PMC8839929; doi:10.3390/plants11030296)
Supplement: Supplementary file 1 [file plants-11-00296-s001.zip › plants-1558469-supplementary.pdf]

Table S1: Primers used in this study

Gene	Function	Forward Primer (5′-3′)	Reverse primer (5′-3′)
EF1α	elongation factor 1-*alpha*	AACCAAAATATCCGGAGTAAAAGA	GAACTGGGTGCTTGATAGGC
60RSP	60S ribosomal protein	ATCTACCTCAAGCTCCTAGTC	CAATCTTGTCCTCCTTTCCT
ACO	1-aminocyclopropane carboxylic acid oxidase	AAGGTCAGCAACTACCCTCC	CGCATCGGTGGAACATCAAT
ERF1	ethylene response factor	CGGTGGTGGCGCTCAAGAGG	TGACCCAGAAGAATCAACGGCTCT
Lox9	9-lipoxygenase	CCCTTCTTGGCATCTCCCTTA	TGTTGTGTCCAGGGTCCATTC
PR1	pathogenesis-related 1	GGAGTCCATTAGCACTCCTTTG	CATAATTCTGGGCGTAGGCAG
PR2	β-1,3-glucanase	TCAATGGCTGCAATGGTGC	CGGTCGATGTTGCGAGATTTA
PR3c	acidic class IV chitinase	TCGAATGCGATGGTGGAAA	TCCCCTGTCGAAACACCAAG
NCED2	9-cis-epoxycarotenoid dioxygenase 2	CTCTTGGCCATGTCGGAAGA	CGGAGCTGCTTGTCGAAGTC
VvGIN1	Vacuolar invertase	CCATCTCCATCCCATCGTAACC	GGCTATCCAAGTTTCCAACCAACC
VvHT1	Hexose transporter 1	TCGGAGTGGATGGAGAACCTTG	GACATCACCACCACAAAGAAGGC
VvHT3	Hexose transporter 3	TAATCGAACGGGGATCAAG	CCCCCAGAAATCAATAAAACTC
VvSWEET2a	Sugars Will Eventually be Exported Transporter 2a	CGTTCTCTGTTGTTGCCAGTC	ACCAAGCAGTTTAGGAGAGCA
VvSWEET4	Sugars Will Eventually be Exported Transporter 4	GGCTCGGACTGTGATTGGTA	ACATGCAGTTCATCACTGTGG
